# The 2-hydroxy-3-(4-aryl-1-piperazinyl)propyl Phthalimide Derivatives as Prodrugs—Spectroscopic and Theoretical Binding Studies with Plasma Proteins

**DOI:** 10.3390/ijms23137003

**Published:** 2022-06-23

**Authors:** Aleksandra Marciniak, Aleksandra Kotynia, Dominika Szkatuła, Edward Krzyżak

**Affiliations:** 1 Department of Inorganic Chemistry, Wroclaw Medical University, ul. Borowska 211a, 50-556 Wrocław, Poland; aleksandra.kotynia@umw.edu.pl (A.K.); edward.krzyzak@umw.edu.pl (E.K.); 2 Department of Medicinal Chemistry, Wroclaw Medical University, ul. Borowska 211, 50-556 Wroclaw, Poland; dominika.szkatula@umw.edu.pl

**Keywords:** plasma proteins, spectroscopic methods, phthalimide analogs, phthalimide derivatives, molecular docking

## Abstract

Many publications in databases deal with the interactions of new drugs with albumin. However, it is not only albumin that is responsible for binding pharmaceutical molecules to proteins in the human body. There are many more proteins in plasma that are important for the study of the ADME pathway. Therefore, in this study, we have shown the results of the interactions between the plasma proteins albumin, orosomucoid, and gamma globulins and non-toxic anti-inflammatory phthalimide analogs, which due to the promising obtained results, may be potential candidates in the group of analgesic and anti-inflammatory drugs. Using spectroscopic methods and molecular modeling, we showed that all four tested compounds form complexes with the analyzed proteins. The formation of a complex with proteins raises the pharmacological efficacy of the drug. Therefore, the obtained results could be a step in the study of the pharmacokinetics and pharmacodynamics of new potential pharmaceuticals.

## 1. Introduction

To identify the ADME (absorption, distribution, metabolism, and excretion) pathway for new pharmaceuticals, the study of the interaction between new compounds and plasma proteins is very important. The concentrations of free and bound forms of the pharmaceuticals are closely related to the interaction with proteins such as albumin or orosomucoid [1,2,3]. Therefore, this type of analysis is one of the steps in the study of the pharmacokinetics and pharmacodynamics of new potential drugs.

Albumin is the main transport protein in the human body [4]. It is also the most frequently chosen protein for the study of interactions with new pharmaceuticals. Due to the analogous structure of albumin of human and bovine origins, the latter is often used in this type of analysis because of its lower cost [5,6]. Aromatic and heterocyclic ligands can bind to subdomains IIA and IIIA in the albumin molecule [5]. However, much less work in the literature deals with the interaction of new pharmaceuticals with proteins other than albumin. Yet, many other plasma proteins also have very important functions from a medical and pharmaceutical point of view.

An example of such proteins is gamma-globulins (GG). They are immune globulins that can bind organic compounds, drugs, metabolites, or antigens. The study of the interactions between this protein and pharmaceuticals has an important role in therapeutic drug monitoring because the binding can affect the free drug concentration, thus affecting its distribution in the body and toxicity [7].

Another plasma protein worth attention is orosomucoid (α1-acid-glycoprotein, AAG). Similar to albumin, it is a transport protein that can bind alkaline molecules. Moreover, it belongs to the acute phase proteins [8]. Therefore, its interaction with drugs is important from the pharmacokinetic point of view.

**Figure 1:**
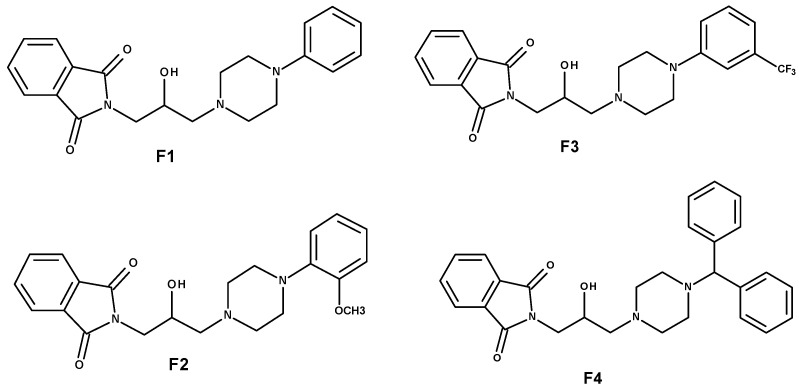
The structures of analyzed compounds F1, F2, F3, and F4.

An interesting group of bioactive molecules is heterocyclic compounds, isoindole derivatives, included in the group of privileged structures [9]. So far, their anti-inflammatory, antibacterial, anticonvulsant, and antifungal properties have been proven [10,11,12]. The definition of the privileged structure describes chemical compounds capable of binding to many biological targets, in line with the principles of modern polypharmacotherapy and current methods of searching for new drugs, which are composed of groups of pharmacophores with an affinity for many different target points of the organism [9]. They are a kind of hybrid of elements determining the desired course of action. The phthalimide pharmacophore, due to the presence of the –CO–N (R) –CO– fragment and an aryl hydrophobic ring, a hydrogen bond donor, electron donors, and other distal substituent donor sites on the imide nitrogen atom, allows it to bind to many biological targets [13,14]. In the structure of the tested F1–F4 derivatives, we combined the isoindole-1,3-dione skeleton and the 2-hydroxy-3-(N-aryl-piperazine)propyl group, which in the case of 3,4-pyridine dicarboxyimides, guaranteed strong analgesic properties, comparable to the action of morphine [15]. The amino residues of the leading structures of the three most active pyrrolo [3.4-c] pyridine-1,3 (2H)dione derivatives (F1–F3) were selected and supplemented with a benzhydryl piperazine substituent (F4) and linked to the phthalimide moiety via a 2-hydroxypropyl linker. The F1–F4 imides were used in behavioral studies on murine analgesic models, which confirmed the supposition that the compounds constructed in this way constitute an interesting group of potential analgesic drugs. In our previous work, we proved the analgesic and anti-inflammatory properties of four new phthalimide derivatives, F1–F4, in in vitro and in vivo studies (Figure 1). Moreover, the non-toxicity of these compounds was shown [16]. Due to such promising results, we decided to continue working on the tested compounds F1–F4.

In this study, we analyzed the interaction between phthalimide derivatives and plasma proteins described above: BSA, GG, and AAG. We used several spectroscopic methods: circular dichroism (CD) spectroscopy, fluorescence spectroscopy, and ATR-IR spectroscopy. Furthermore, molecular modeling methods were used to investigate all analyzed interactions. The results obtained by all theoretical and analytical methods used allow the determination of whether the analyzed phthalimide derivatives bind to and interact with the studied proteins.

## 2. Results and Discussion

### 2.1. Fluorescence Spectroscopy

#### 2.1.1. Fluorescence Quenching of BSA, AAG, and GG in the Presence of Compounds F1–F4

**Figure 2:**
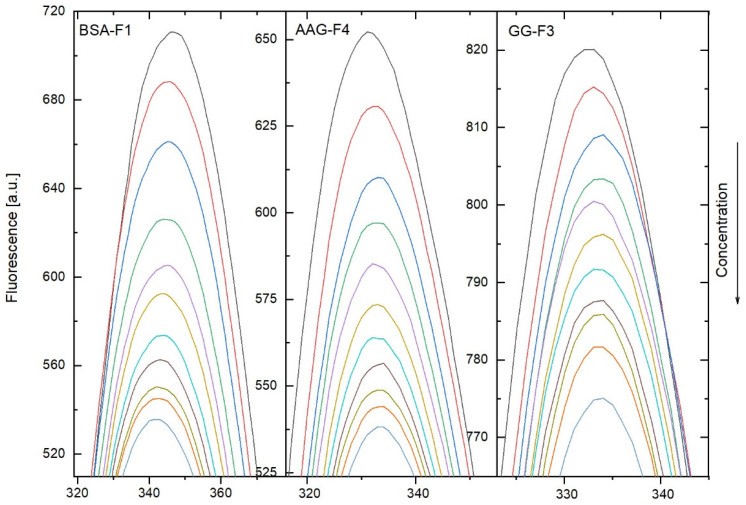
Fluorescence spectra (zoom on maximum) of BSA-F1, AAG-F4, and GG-F3 systems in the presence of compounds F1, F4, and F3. The concentrations were: 0, 0.2, 0.4, 0.6, 0.8, 1.0, 1.2, 1.4, 1.6, 1.8, and 2.0 µM.

A fluorescence quenching experiment was performed to define the nature of the interactions between the studied compounds and bovine serum albumin (BSA), α1-acid glcprotein (AAG), and gamma globulin (GG). The excitation wavelength was selected as 280 nm. The conformation changes were evaluated by measuring the intrinsic fluorescence intensity of the protein Trp and Tyr residues before and after the addition of compounds F1–F4. From the experimental observations, the fluorescence intensity of BSA, AAG, and GG was found to decrease regularly with the increasing concentration of F1–F4. This result suggests an interaction with the studied compounds. The selected quenching spectra of BSA, AAG, and GG after the addition of the tested compounds are presented in Figure 2 (zoom to the position of the maximum emission). The fluorescence plot in the whole range is presented in the supplementary file (Figure S1). The spectra for the other studied systems are also included in the supplementary file (Figures S2–S7). A shift in the maximum emission was observed. For the interaction with BSA, the peak was shifted to a shorter wavelength. This means that the amino acid residues were located in a more hydrophobic environment and were less exposed to the solvent [17]. For the interaction with AAG and GG, a small red shift was detected. It indicates that the conformations of AAG and GG were changed and that the amino acid residues were in a more polar environment.

#### 2.1.2. Quenching Mechanism Analysis

Fluorescence quenching and a shift of the maximum emission peak can suggest a static quenching mechanism and the formation of stable complexes. However, it can also be the result of collisional encounters (dynamic quenching). To confirm the quenching mechanism and complex formation, the fluorescence data were further analyzed by the Stern–Volmer equation and dependence on temperature. The fluorescence intensities were corrected by Equation (1):

(1) $$F_{\mathrm{corr}}=F_{\mathrm{obs}}10^{\frac{\left(A_{\mathrm{ex}}+A_{\mathrm{em}}\right)}{2}}$$

where *F_corr_* and *F_obs_* are the corrected and observed fluorescence intensities, respectively, and *A_ex_* and *A_em_* are the absorbance values at the excitation and emission wavelengths, respectively. 

The possible quenching mechanism was examined using the classical Equation (2):

(2) $$\frac{F_{0}}{F}=1+k_{q}\tau \left[Q\right]=1+K_{\mathrm{SV}}$$

where *F*_0_ and *F* are the steady-state fluorescence intensities at the maximum wavelength in the absence and presence of a quencher, respectively, *k_q_* is the quenching rate constant of the biomolecule, *τ*_0_ is the average lifetime of the biomolecule, [*Q*] is the quencher concentration, and *K_SV_* is the Stern–Volmer constant. 

The measurements were carried out at three temperatures: 297, 303, and 308 K. The Stern–Volmer (*K_SV_*) constant was determined by linear fitting. The calculated results are collected in Table 1, Table 2 and Table 3.

The results showed that obtained k_q_ values at a temperature of 297 K were from 0.29 ot 1.77 × 10^13^ for the interaction with BSA, from 6.24 to 22.54 × 10^11^ for the interaction with AAG, and from 5.73 to 31.80 × 10^11^ for the interaction with GG. For dynamic quenching, the maximum scatters collision quenching constant of numerous quenchers with the biopolymers was reported to be 2 × 10^10^ dm^3^·mol^−1^·s^−1^ [18,19], which suggests the formation of complexes for all analyzed compounds, F1–F4, with the studied proteins. To confirm this, the temperature dependence of k_q_ was investigated. The obtained results are listed in Table 1, Table 2 and Table 3. The K_SV_ and k_q_ values decreased with increasing temperatures. This also indicates the static quenching mechanism.

#### 2.1.3. Binding Constant and Thermodynamic Parameters

**Figure 3:**
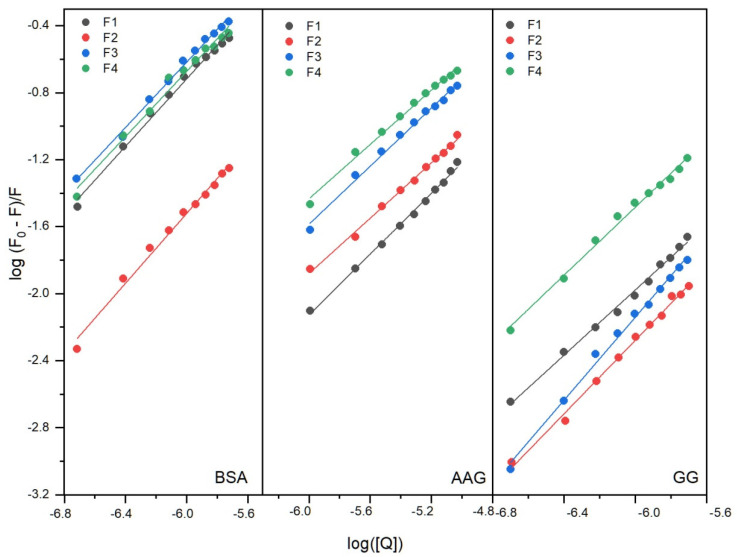
Double logarithm regression plots for quenching of BSA, AAG, and GG by compounds F1–F4.

Binding constants and the number of binding sites were obtained from Equation (3):

(3) $$\log\frac{F_{0}-F}{F}=\log K_{b}+\mathrm{nlog}\left[Q\right]$$

where *F*_0_ and *F* are the steady-state fluorescence intensities at the maximum wavelength in the absence and the presence of a quencher, respectively, and [*Q*] is the quencher concentration. The fluorescence quenching data were plotted as log [(*F*_0_ − *F*)/*F*] vs. *log*[*Q*] and are shown in Figure 3.

A good linear fit was observed. The values of the binding constant (K_b_) and the number of binding sites (n) were calculated by the slope and intercept of Equation (3). The results are listed in Table 1, Table 2 and Table 3. The most stable complexes were formed from the interactions of the studied compounds with BSA. For the BSA-F1 complex, the value of K_b_ was found to be 2.13 × 10^5^ dm^3^·mol^−1^. Structural modification slightly reduced the K_b_ value, at least when one benzene ring was replaced with two (compound F4). In the case of introducing a substituent in the phenyl (compounds F2 and F3), the K_b_ values were found to be 0.55 × 10^5^ and 1.75 × 10^5^ dm^3^·mol^−1^, respectively. The number of binding sites in all systems was close to 1, which shows one-to-one interactions. The cmpounds tested in this work were designed with potential analgesic and anti-inflammatory effects [16]. F. Mohammadnia investigated the interaction of 14 anti-inflammatory drugs with human serum albumin [20]. The binding constants were found to be in the range of 10^2^–1.88 × 10^7^ dm^3^·mol^−1^ for acetaminophen and meloxicam as drugs with borderline values. Therefore, the K_b_ values of the studied compounds show that the interactions with BSA fall within this range. Similar values were obtained for many compounds with biological activity [21,22,23,24,25,26,27]. For the interactions with AAG, the binding constants (K_b_ values) were found in the range from 1.22 × 10^3^ to 4.1 × 10^3^ dm^3^·mol^−1^ (Table 2). Compound F3 with an additional –CF_3_ group in the phenyl formed the most stable complex. Replacing one benzene ring with two (compound F4) also increased the stability relative to F1, whereas the additional –OCH_3_ group in the phenyl slightly lowered the K_b_ value. For the systems with GG, the stability of complexes increased in the order of F1–F2 (–OCH_3_ group in an aromatic ring), F4 (two benzene rings), and F3 (–CF_3_ group in an aromatic ring). The binding constants, K_b,_ were greater than for AAG systems (Table 2 and Table 3). However, the results indicated that complexes formed with both AAG and GG were weaker than for BSA. It can be concluded that both proteins would be involved in the distribution of F1–F4 in the bloodstream to a smaller extent. On the other hand, the lower K_b_ values indicate an easier release of F1–F4 from the complex. Anyway, the formation of a complex with all studied proteins from the plasma raises the pharmacological efficacy of the drug [8,28]. 

Molecules can interact with proteins by hydrogen bond, van der Waals force, electrostatic and hydrophobic interactions, etc. [29]. The values of the thermodynamic parameters of enthalpy change (ΔH°), entropic change (ΔS°), and free energy change (ΔG°) indicate the types of these interactions. The thermodynamic parameters were calculated from Equations (4) and (5):

(4) $$\log K_{b}=-\frac{\Delta H^{{}^{\circ}}}{RT}+\frac{\Delta S^{{}^{\circ}}}{R}$$

(5) $$\Delta G^{{}^{\circ}}=\Delta H^{{}^{\circ}}-T\Delta S^{{}^{\circ}}=-\mathrm{RTln}K_{b}$$

where K_b_ is the binding constant and R is the universal gas constant. 

The calculated results are given in Table 1, Table 2 and Table 3. The results showed that the binding interactions between compounds F1–F4 and all studied proteins were spontaneous due to the negative ΔG° values. Moreover, both the ΔH° and ΔS° negative values indicated that the main interaction force in the binding process was van der Waals forces and/or hydrogen bonding interactions.

It is known that BSA as well as human serum albumin (HSA) have two binding sites (site I and site II), which are located in subdomains IIA and IIIA, respectively [30]. To evaluate the binding site in BSA for F1–F4, fluorescence quenching was carried out by using phenylbutazone (PHB) and ibuprofen (IBP) as site probes. Site I showed binding affinity towards PHB, and site II is known to bind IBP [31]. The results are summarized in Table 4. LogK_b_ was calculated using Equation (3). The results show that the K_b_ value for the interactions with all tested compounds decreased in the presence of both PHB and IBP markers. However, for PHB, the differences were much smaller than in IBP. To sum up, compounds F1–F4 may bind to subdomain IIA or IIIA of BSA. However, it seems that site II is much more preferred.

### 2.2. Circular Dichroism Spectroscopy

**Figure 4:**
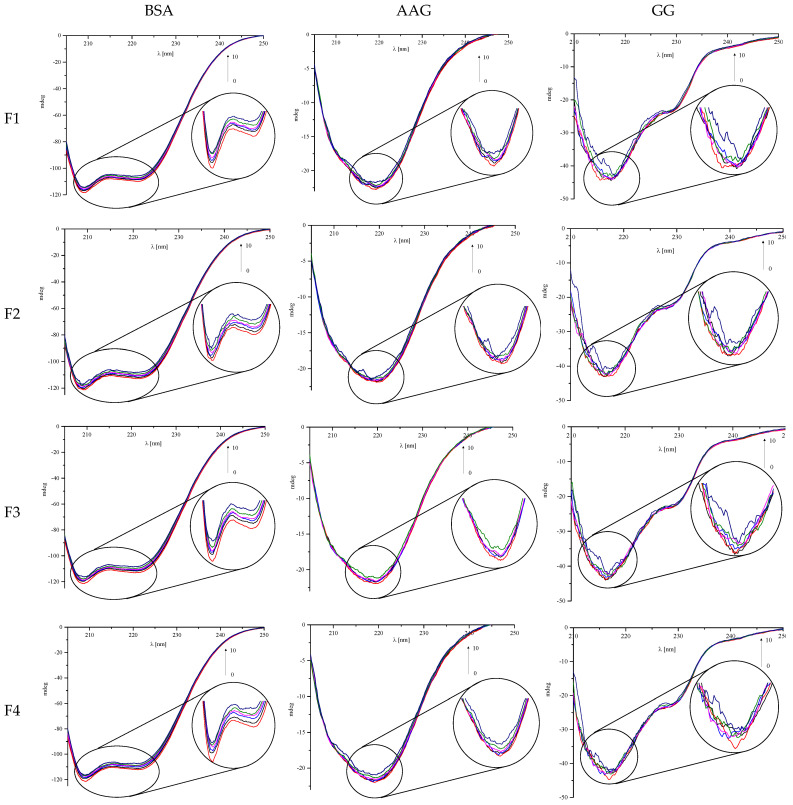
Circular dichroism spectra of BSA, AAG, and GG in the absence and presence of analyzed phthalimide derivatives F1, F2, F3, and F4.

The observation of the secondary structure of proteins can be successfully performed using circular dichroism (CD) spectroscopy [32]. On the CD spectrum, the characteristic bands of specific secondary structures can be observed. Two negative peaks near 209 and 220 nm are typical for the α-helix, while the negative band around 215 can be attributed to the presence of the β-sheet [33]. We measured the CD spectra for all analyzed proteins in the absence and presence of phthalimide derivatives F1–F4 (Figure 4). We wanted to observe the changes after adding every portion of the analyzed molecules, from 1:0 to 1:10 molar ratios of protein to phthalimide derivatives. The changes in the recorded CD spectra can confirm the formation of complexes between the proteins and the test compounds. To calculate the percentage of secondary structure elements of proteins, the obtained spectra were analyzed by the CD Multivariate SSE program. The obtained results are summarized in Tables S1–S3.

For BSA, two negative bands were observed (Figure 4) near 209 and 220 nm. They were characteristic of the α-helix structure. It can be concluded that the tested compounds interacted with the albumin molecule because the addition of each successive portion of the analyzed phthalimide derivative reduced the intensity of both bands. However, they did not destabilize the protein structure. The alpha-helix was still the dominant form of the protein, even after adding a 10-fold excess of the test compounds (Table S1). The observed changes in the content of α-helix were the largest for compound F3 (2.6%), and the smallest for F1 (1.9%).

AAG had one negative band near 220 nm on the CD spectra (Figure 4). The addition of each successive portion of the analyzed phthalimide derivatives did not cause significant changes in the course of the spectra. The structure of the protein consisted of about 30% α-helix and β-sheet (Table S2). The percentage of α-helix slightly decreased after the addition of subsequent portions of the analyzed compounds, while the quantity of β-sheet increased. The maximum changes were for F1 and F4 (1.7 % for α-helix, and 0.5 and 1.0 for β-sheet, respectively). Interestingly, virtually no changes were observed for F3. Therefore, it can be concluded that the binding of phthalimide derivatives to AAG does not affect its structure.

In the case of GG, the greatest changes in the course of the CD spectra were observed after adding subsequent portions of the tested phthalimide derivatives (Figure 4). Each subsequent portion of the analyzed compound caused a greater noise in the spectrum. However, as can be seen in Table S3, it did not significantly affect the percentage of the various forms. Even after adding a 10-fold excess of the analyzed compounds, no significant changes were seen.

### 2.3. The ATR-IR Spectroscopy 

**Figure 5:**
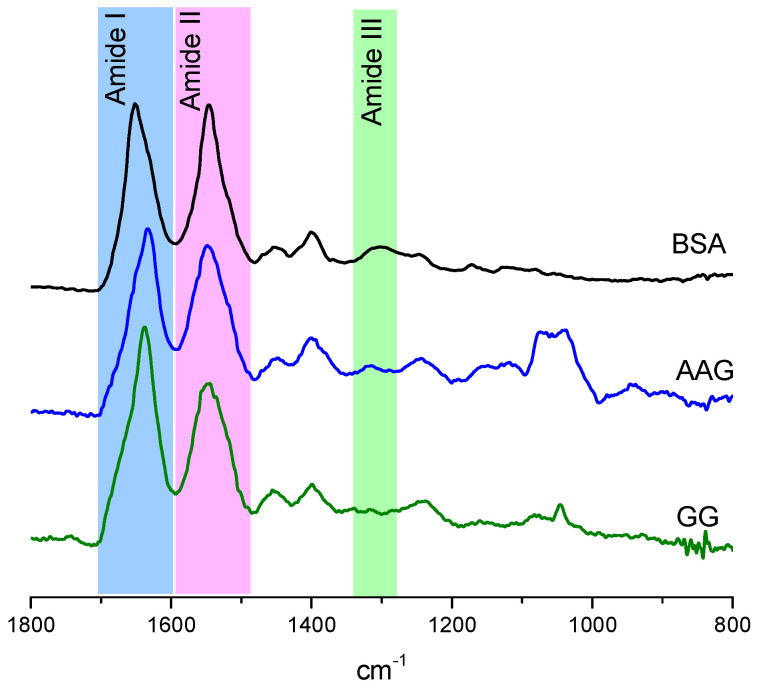
The ATR-IR spectra of fingerprint range for BSA (black line), AAG (blue line), and GG (green line).

To understand the changes in the secondary structures of the blood plasma proteins, the ATR-IR analysis was performed. The spectra of solutions with the free proteins BSA, AAG, and GG were recorded, and the most sensitive range of the fingerprint for each of them is presented in Figure 5. The characteristic peaks for protein were detected and identified as amides I, II, and III, which are distinguished as color areas in Figure 5. The details of all IR signals are collected in Table 5. The most diverse was the position of the amide I, which was 1651 cm^−1^ for BSA, whereas it was 1634 cm^−1^ for AGG and 1639 cm^−1^ for GG. On the other hand, for the amide II band, which was found at 1645 cm^−1^ (for BSA) and 1648 cm^−1^ (for AAG and GG), no such large differences in position were observed. The amide III was present at 1301 cm^−1^ for BSA and at 1318 cm^−1^ for AAG but was absent for GG. The amide I signal was primarily assigned to N–H stretching vibration in about 80% of the contribution. However, the C–N stretching vibration and N–H bending vibration were also included. The amide II was caused mainly by the N–H bending and C–N stretching vibration, similar to the amide III band [34,35].

**Figure 6:**
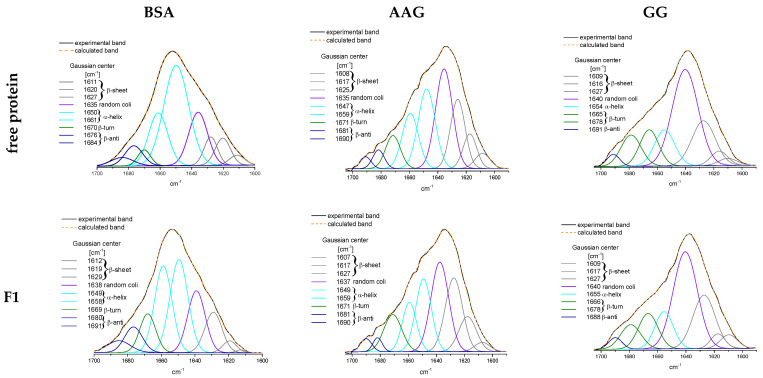

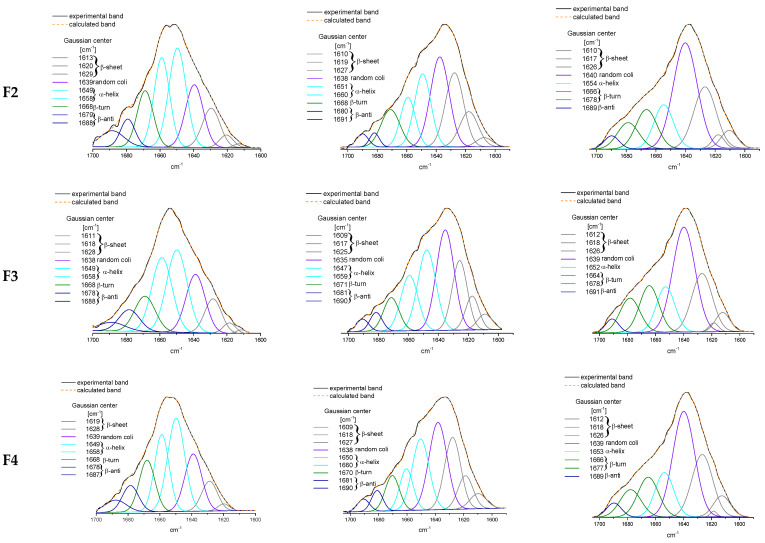
The deconvolution of amide I band of blood serum proteins BSA, AAG, and GG and complexes with compounds F1–F4, obtained by ATR-IR spectra.

Generally, in infrared spectra, the amide bands are primarily assigned to the varied secondary structural compositions of proteins. The most common choice of quantitative analysis of the contribution of individual structures of the secondary protein structure is the deconvolution of the amide I band, but sometimes the deconvolution of amide II or amide III could give a rewarding result [35,36,37,38,39,40]. It is a very useful method to examine a new class of compounds such as prodrugs to determine the interaction with blood plasma proteins [41,42,43]. Obviously, this is only the beginning of studying the ADME pathways. The amide I band is a composite of overlapping component bands that represent different structural elements. The second derivative enabled the differentiation of major peaks whose positions are responsible for the α-helix (1660–1650 cm^−1^), β-sheet (1640–1610 cm^−1^), β-turn (1691–1680 cm^−1^), β-antiparallel (1660–1650 cm^−1^), and random coil (1650–1640 cm^−1^) structures [44,45,46]. The area under each peak directly corresponds to the percentage of the protein structure. The self-deconvolution of amide I peak and Gaussian function fitting were carried out, and the results are presented in Table S4 and Figure 6. 

The binding of the studied phthalimide derivatives to all proteins has brought down their α-helix structure (Table S4, Figure 6). It was apparently manifested for the BSA where the percentage of α-helix was reduced by about 10–12%. At the same time, an increase in the share of β-sheet and the random coil was observed. In the case of the complexation of F1–F4 to AAG, this resulted in a reduction in α-helix by an average of about 6% and a slightly lower (~2%) random coil for increased β structures (sheet, turn, and antiparallel). The addition of phthalimide derivatives very slightly modified the signal of amide I for GG, supporting the hypothesis that the protein conformation was still essentially the same before and after compound binding. The overall conclusion is that F2, which demonstrated the –OCH3 group in the phenyl, had the strongest influence on the structures of all proteins.

### 2.4. Molecular Docking Studies

To determine the preferred binding location and type of interactions of compounds F1–F4 with BSA, AAG, and GG, the molecular docking method was used. The simulated results are presented in Table 6.

**Figure 7:**
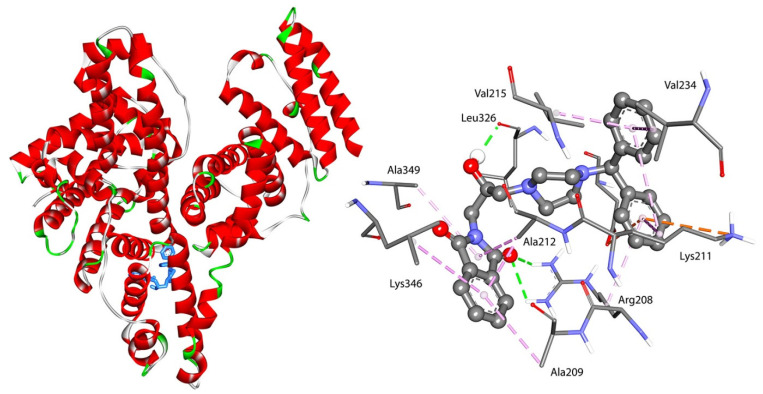
Docked pose and interactions of compound F4 with BSA in site II.

All studied compounds had a good affinity to interact with selected plasma proteins. The binding free energy (ΔG°) for interactions was found to be negative. It indicated the formation of stable complexes. As is well known, the more negative the binding free energy ΔG°, the more stable the complex that is formed. For the BSA system, the results revealed that the binding free energy for F1–F4 within the hydrophobic cavity in site II (subdomain IIIA) was more negative than that within the hydrophobic cavity in site I (subdomain IIA). This indicates that site II was favorable. The lowest (more negative) energy was found for F4, with two benzene rings. The position of compound F4 in the pocket site II of BSA is given in Figure 7.

**Figure 8:**
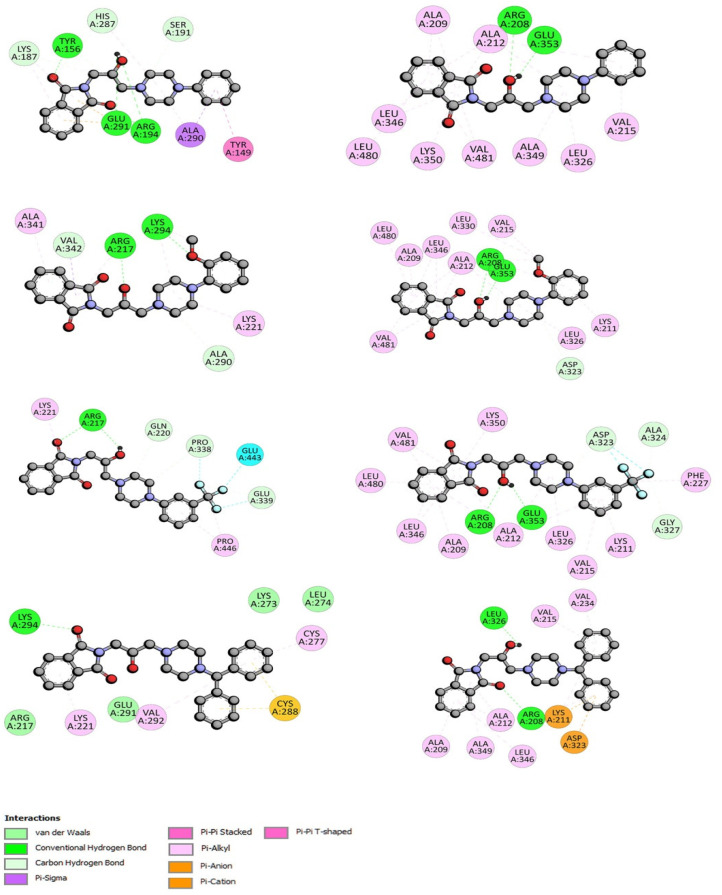
The 2D interaction plots of compounds F1–F4 with BSA in site I (**left**) and site II (**right**).

Two hydrogen bonds were formed between Arg208 and the oxygen atom from the carbonyl group and between Leu326 and the –OH from the linker. The molecule was also stabilized by π-alkyl interactions via the isoindoline-1,3-dione moiety and the Ala209, Ala212, Ala349, and Leu346 residues and via benzene rings with Val215, Val234, and Lys211. The π-cation contacts with Lys211 and π-anion with Asp323 were observed. The two hydrogen bonds were also formed for the interactions of compounds F1–F3 with BSA into site II. The hydroxy group from the linker, Arg206, and Gly353 are involved. The π-alkyl interactions were observed. The details are presented in Figure 8. In site I, the studied compounds interacted with BSA via hydrogen bonds between the carbonyl or hydroxy group and Tyr156, Arg194, Arg217, Glu291, and Lys294. Besides hydrogen bonds, many types of hydrophobic interaction were observed. The details are presented in Figure 8 (left). The most stable complex formed compound F4, just like in site I. 

**Figure 9:**
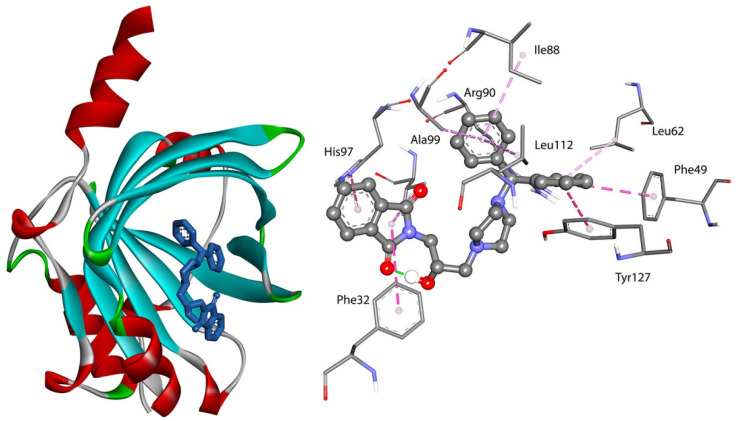
Docked pose and interactions of compound F4 with AAG.

**Figure 10:**
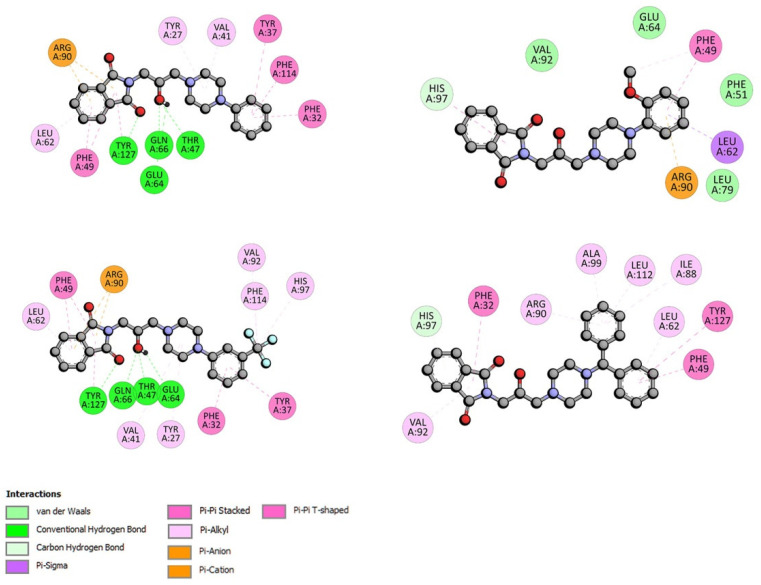
The 2D interaction plots of compounds F1–F4 with AAG.

For systems with AAG, all compounds formed a stable complex. The binding free energy (ΔG°) was negative and took values in the range of −33 to −39 kJmol^−1^ (Table 6). The obtained values were slightly smaller than in the case of interactions with BSA. The best result was calculated for the AAG-F4 complex, just like for the BSA system. The position of compound F4 and the interactions with AAG are presented in Figure 9. No hydrogen bonds were observed. Two benzene rings interacted with the protein via hydrophobic contacts: π-π stacked with Phe49, Tyr217, π-alkyl with Leu62, Ile88, Arg90, Ala99, and Leu112. The isoindoline-1,3-dione moiety was involved in a π-π shaped contact with Phe32, and π-alkyl was involved with Val92. The carbonyl group formed an isoindoline-1,3-dione moiety, and the –OH group from the linker played an important role in the stability of the complex with AAG and also for the other tested compounds. The hydrogen bonds with Thr47, Glu64, Gln66, and Tyr127 were formed. Various kinds of hydrophobic contacts with the isoindoline-1,3-dione moiety, piperazine ring, and benzene ring were observed. The details are presented in Figure 10.

**Figure 11:**
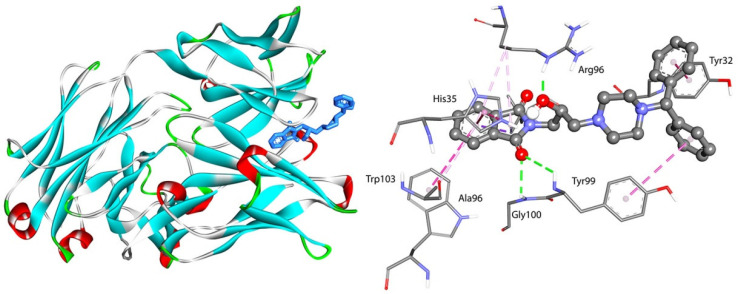
Docked pose and interactions of compound F4 with GG.

**Figure 12:**
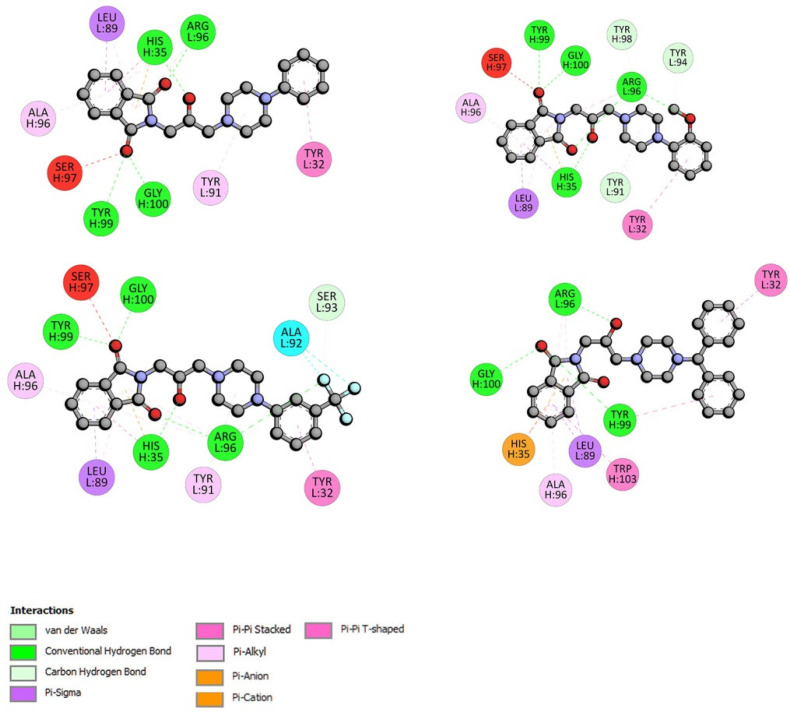
The 2D interaction plots of compounds F1–F4 with GG.

For interactions with GG, the ΔG° was found to be from −33.83 kJmol^−1^ for the GG-F3 complex to −35.38 kJmol^−1^ for the GG-F4 complex. These values were similar to those calculated for the interactions with AAG, except for F4 (with two benzene rings). The pose of compound F4 and the interactions with GG are presented in Figure 11. Three hydrogen bonds were observed between Tyr99, Gly100, and the oxygen atom from the carbonyl group and between Arg96 and the –OH from the linker. The various types of π contacts were formed. Compounds F1–F3 also bonded to GG by hydrogen bonds between the isoindoline-1,3-dione moiety, linker, Arg96, Tyr100, His35, Gly100, and Tyr99. The details are given in Figure 12.

## 3. Materials and Methods

### 3.1. Chemicals

The synthesis of the analyzed compounds was performed in the Department of Medicinal Chemistry, Wroclaw Medical University, and was described in our previous work [16]. Bovine serum albumin (BSA), bovine α1-acid glycoprotein (AAG), bovine gamma globulin (GG), and phosphate buffer were obtained from Sigma-Aldrich Chemie GmbH, (St. Louis, MO, USA). 

### 3.2. Spectroscopic Studies

#### 3.2.1. Fluorescence Spectroscopy

The fluorescence spectroscopy measurements were performed using a Cary Eclipse 500 spectrophotometer (Agilent, Santa Clara, CA, USA). The concentrations of BSA, AAG, and GG were 1.0 × 10^−6^ mol/dm^3^, and 3 cm^3^ of a solution of each protein was titrated by successive additions of 1.0 × 10^−3^ mol/dm^3^ solutions of the studied compounds, F1–F4, to give a final concentration of 0.2 × 10^−6^–2.0 × 10^−6^ mol/dm^3^. Experiments were carried out at three temperatures: 297, 303, and 308 K in phosphate buffer as a solvent (pH 7.5). The quenching spectra were recorded at an excitation equal to 280 nm and an emission wavelength of 300–500 nm with a 10 mm path length. The molar ratio of compound to protein was 0.1–2.0 with 0.2 steps for BSA and GG and 1–10 with 1.0 steps for AGG. Furthermore, binding site identification studies for BSA were indicated in the presence of the two site markers: phenylbutazone (PHB) and ibuprofen (IBP), as site I and II markers, respectively. The concentrations of BSA and site markers were 1.0 × 10^−6^ and 3.0 × 10^−6^ mol/dm^3^, respectively.

#### 3.2.2. Circular Dichroism Spectroscopy

Circular dichroism (CD) spectra were measured on a Jasco J-1500 magnetic circular dichroism spectrometer (JASCO International CO., Tokyo, Japan). All of the measurements for the BSA, AAG, and GG solutions in the absence and presence of the analyzed compounds were made at room temperature under simulated physiological conditions at pH 7.4 in phosphate buffer as a solvent. The CD spectra were measured in the range of 205–250 nm for BSA and AAG and 210–250 for GG at a scan rate speed of 50 nm/min with a response time of 1 s and a 10 mm path length. All spectra were baseline-corrected. The concentrations of BSA, AAG, and GG were 1 × 10^−6^ mol/dm^3^. For the analyzed compounds F1, F2, F3, and F4, the concentration was equal to 1×10^−3^ mol/dm^3^. Experiments were performed on each analyzed compound in molar protein-to-ligand ratios equal to 1:0, 1:0.5, 1:1, 1:5, and 1:10, and 2 cm^3^ of a solution of each protein was titrated by successive additions of solutions of the analyzed compounds to give final molar ratios. The percentage content of the secondary structure elements of the analyzed proteins was obtained by CD Multivariate Calibration Creation and CD Multivariate SSE programs (JASCO International CO., Tokyo, Japan). For this purpose, the protein concentrations were converted to mean residue molar concentrations.

#### 3.2.3. ATR-IR Spectroscopy

A Nicolet iS50 FT-IR (Thermo Fisher Scientific Waltham, MA, USA) was used to record the spectra of the attenuated total reflectance infrared spectroscopy (ATR-IR). The spectrometer was equipped with a deuterated triglycine sulfate (DTGS) detector and a KBr beam splitter. The spectra were recorded in the range of 3000–600 cm^−1^ with a resolution 4 cm^−1^, and 100 scans for each spectrum were performed. The data analysis was conducted with Omnic 9.3.30 (Thermo Fisher Scientific Inc.) software.

The samples were prepared by mixing a protein solution with the phthalimide solution to obtain the equimolar ratio at room temperature. The concentration of BSA (Sigma Aldrich Chemie GmbH, St. Louis, MO, USA) was 1 × 10^−3^ mol/dm^3^, and the concentrations of AAG and GG (Sigma Aldrich Chemie GmbH, St. Louis, MO, USA) were each 1 × 10^−5^ mol/dm^3^. All proteins were dissolved in phosphate buffer (pH = 7.5) (Sigma Aldrich, Chemie GmbH, St. Louis, MO, USA). The concentration of compounds F1, F2, F3, and F4 was 1 × 10^−2^ mol/dm^3^ for measuring the BSA interaction and 1 × 10^−4^ mol/dm^3^ for measuring the AAG and GG interactions.

### 3.3. Molecular Docking

The crystal structures of serum albumin (3V03), α1-acid glycoprotein (3KQ0), and gamma globulin (1AJ7) were obtained from the Protein Data Bank (Available online: http://www.rcsb.org (accessed on 5 February 2022)). The structures of the studied compounds were optimized using DFT functional with the B3LYP/6-311+G (d.p) basic set [47,48,49]. Calculations were carried out using the Gaussian 2016 A.03 software package [50]. The molecular docking study was conducted using AutoDock 4.2.6 software and AutoDock Tools 1.5.6 [51]. All the ligands and water molecules were removed, then polar hydrogen atoms and Kollman charges were added to the protein structure. To prepare the ligand molecules, partial charges were calculated, nonpolar hydrogens were merged, and rotatable bonds were assigned. The Lamarckian genetic algorithm was selected for the conformational search. The running times of the genetic algorithm and the evaluation times were set to 100 and 2.5 million, respectively. The centers of the grid boxes were set according to the ligand’s binding site in the crystal structure. After the molecular docking, the ligand–receptor complexes were further analyzed using Discovery Studio Visualizer v.20 (Available online: https://www.3ds.com/ accessed on (5 February 2022)).

## 4. Conclusions

In this study, we showed that all four analyzed phthalimide derivatives could form stable complexes with the studied plasma proteins: albumin, orosomucoid, and gamma globulin. The results obtained by fluorescence spectroscopy confirmed the static quenching mechanism and the formation of one-to-one complexes. The most stable interactions were identified for the analyzed molecules and BSA, especially F1-BSA, that is, for a derivative with no substituents. Both the fluorescence spectroscopy and docking studies showed that it is more likely to bind to site II in the albumin molecule. Compound F2 with an –OCH_3_ group formed the weakest complex. The complexes with AAG and GG were weaker than with BSA. However, of these two proteins, the former was more strongly bound to the tested derivatives. For both of these proteins, the most stable complexes were formed with the F3 derivative, with a CF_3_ group. The CD spectroscopy results showed that the formation of complexes does not destabilize the protein structures. The greatest changes in the course of the CD spectra were observed in the case of GG. However, they did not significantly affect the percentages of the various forms of secondary structures of proteins. Generally, the formation of a complex with proteins from plasma raises the pharmacological efficacy of a drug. A previous study showed that the compounds are non-toxic and could potentially be anti-inflammatory and analgesic pharmaceuticals. Therefore, in connection with the results obtained in this manuscript, it can be concluded that the studied phthalimide analogs are a very promising tool for searching for new drugs.

## Figures and Tables

**Table 1:** The Stern–Volmer constant, K_sv_, quenching rate constant, k_q_, binding constant, K_b_, number of binding sites, n, and thermodynamic parameters for the interaction of BSA with the studied compounds at different temperatures.

		Quenching		Binding			Thermodynamic		
	T (K)	K_sv_ × 10^5^ [dm^3^·mol^−1^]	k_q_ × 10^13^ [dm^3^·mol^−1^·s^−1^]	logK_b_	K_b_ × 10^5^ [dm^3^·mol^−1^]	n	ΔG° (kJmol^−1^)	ΔH° (kJmol^−1^)	ΔS° (Jmol^−1^K^−1^)
F1	297 303 308	1.77 1.76 1.50	1.77 1.76 1.50	5.33 ± 0.20 5.04 ± 0.24 3.82 ± 0.20	2.13 1.10 0.61	1.00 ± 0.03 0.97 ± 0.04 0.93 ± 0.08	−30.33	−85.86	−186.98
F2	297 303 308	0.29 0.27 0.12	0.29 0.27 0.12	4.74 ± 0.23 4.21 ± 0.40 3.96 ± 0.14	0.55 0.16 0.09	1.04 ± 0.03 0.97 ± 0.06 0.97 ± 0.02	−26.79	−125.30	−331.64
F3	297 303 308	2.30 2.07 1.50	2.30 2.07 1.50	5.24 ± 0.14 4.93 ± 0.08 4.83 ± 0.17	1.75 0.85 0.68	0.98 ± 0.02 0.93 ± 0.01 0.94 ± 0.02	−29.67	−66.22	−123.04
F4	297 303 308	1.93 1.00 0.89	1.93 1.00 0.89	5.30 ± 0.27 4.70 ± 0.17 4.36 ± 0.06	1.99 0.51 0.27	0.99 ± 0.04 0.95 ± 0.03 0.90 ± 0.01	−30.02	−125.08	−410.96

**Table 2:** The Stern–Volmer constant, K_sv_, quenching rate constant, k_q_, binding constant, K_b_, number of binding sites, n, and thermodynamic parameters for the interaction of AAG with the studied compounds at different temperatures.

		Quenching		Binding			Thermodynamic		
	T (K)	K_sv_ × 10^3^ [dm^3^·mol^−1^]	k_q_ × 10^11^ [dm^3^·mol^−1^·s^−1^]	logK_b_	K_b_ × 10^3^ [dm^3^·mol^−1^]	n	ΔG° (kJmol^−1^)	ΔH° (kJmol^−1^)	ΔS° (Jmol^−1^ K^−1^)
F1	297 303 308	6.24 4.39 3.19	6.24 4.39 3.19	3.33 ± 0.10 3.01 ± 0.20 2.74 ± 0.19	2.12 1.02 0.55	0.91 ± 0.02 0.87 ± 0.04 0.85 ± 0.03	−18.88	−92.31	−274.25
F2	297 303 308	8.85 2.62 1.52	8.85 2.62 1.52	3.08 ± 0.10 2.65 ± 0.20 1.89 ± 0.24	1.21 0.44 0.08	0.83 ± 0.02 0.84 ± 0.03 0.75 ± 0.04	−17.87	−186.90	−569.17
F3	297 303 308	18.25 9.93 4.40	18.25 9.93 4.40	3.62 ± 0.13 3.16 ± 0.07 2.49 ± 0.15	4.17 1.44 0.31	0.87 ± 0.02 0.83 ± 0.01 0.77 ± 0.03	−20.85	−177.97	−529.02
F4	297 303 308	22.54 13.99 4.37	22.54 13.99 4.37	3.44 ± 0.13 3.06 ± 0.03 2.34 ± 0.08	2.75 1.16 0.22	0.81 ± 0.02 0.78 ± 0.01 0.74 ± 0.02	−19.93	−172.46	−513.66

**Table 3:** The Stern–Volmer constant, K_sv,_ quenching rate constant, k_q_, binding constant, K_b,_ number of binding sites, n, and thermodynamic parameters for the interaction of GG with the studied compounds at different temperatures.

		Quenching		Binding			Thermodynamic		
	T (K)	K_sv_ × 10^3^ [dm^3^·mol^−1^]	k_q_ × 10^11^ [dm^3^·mol^−1^·s^−1^]	logK_b_	K_b_ × 10^3^ [dm^3^·mol^−1^]	n	ΔG° (kJmol^−1^)	ΔH° (kJmol^−1^)	ΔS° (Jmol^−1^K^−1^)
F1	297 303 308	10.77 6.83 5.25	10.77 6.83 5.25	3.87 ± 0.15 3.57 ± 0.20 3.35 ± 0.11	7.38 3.71 2.26	0.97 ± 0.03 0.95 ± 0.03 0.93 ± 0.02	−21.93	−81.27	−199.7
F2	297 303 308	5.73 4.36 3.81	5.73 4.36 3.81	4.31 ± 0.17 4.01 ± 0.12 3.97 ± 0.14	20.53 10.32 9.26	1.09 ± 0.03 0.89 ± 0.02 0.89 ± 0.02	−24.31	−55.40	−104.67
F3	297 303 308	8.28 5.91 5.65	8.28 5.91 5.65	5.30 ± 0.19 4.91 ± 0.16 4.19 ± 0.24	199.8 81.43 15.50	1.24 ± 0.03 1.20 ± 0.03 1.08 ± 0.04	−30.52	−174.11	−483.46
F4	297 303 308	31.80 27.04 16.71	31.80 27.04 16.71	4.61 ± 0.18 4.31 ± 0.07 3.97 ± 0.10	41.04 20.34 9.33	1.02 ± 0.03 0.98 ± 0.02 0.96 ± 0.02	−26.41	−104.54	−263.06

**Table 4:** The binding constants of the studied compounds with BSA in the presence of site markers: phenylbutazone (PHB) and ibuprofen (IBP) at 297 K.

Site Marker	logK_b_			
	F1	F2	F3	F4
**BSA**	5.33 ± 0.20	4.74 ± 0.23	5.23 ± 0.14	5.30 ± 0.27
**BSA + PHE (site I)**	4.96 ± 0.17	4.55 ± 0.07	5.09 ± 0.26	5.08 ± 0.24
**BSA + IBP (site II)**	3.47 ± 0.19	3.55 ± 0.19	3.17 ± 0.16	3.90 ± 0.32

**Table 5:** The characteristic absorption band was detected in ATR-IR spectra of proteins.

Wavenumber Band cm^−1^			Assignments
BSA	AAG	GG	
1651	1634	1639	Amide I—ν(C=O), ν(C–N), δ(N–H)
1545	1548	1548	Amide II—δ(N–H), ν(C–N)
1450	1452	1452	δ_s_(CH_3_), δ_as_(CH_3_)
1399	1402	1401	ν_s_(COO^-^)
1301	1318	-	Amide III—ν(C–N), δ(C–N), δ(O=C–N)
1246	1245	1240	ν(C–O), δ(C–H_2_)
1173	1152	1048	ν(C–O)
	1121		ν(C–O)

ν—stretching vibration, δ—bending vibration, s—symmetric, as—asymmetric.

**Table 6:** Binding Free Energy ΔG° complexes F1–F4 with BSA, AAG, and GG.

Binding Free Energy ΔG° [kJmol^−1^]				
Compound	BSA		AAG	GG
	Site I	Site II		
F1	−30.72	−36.44	−34.31	−34.00
F2	−28.88	−34.99	−33.23	−34.97
F3	−27.50	−36.40	−34.02	−33.83
F4	−34.06	−40.71	−39.28	−35.38

## Data Availability

Not applicable.

## Supplementary Materials

The following supporting information can be downloaded at: www.mdpi.com/article/10.3390/ijms23136999/s1.
