# Supplementary material for: The 2-hydroxy-3-(4-aryl-1-piperazinyl)propyl Phthalimide Derivatives as Prodrugs—Spectroscopic and Theoretical Binding Studies with Plasma Proteins
Source: Int J Mol Sci. 2022 Jun 23;23(13):7003. doi: 10.3390/ijms23137003 (PMC9266550; doi:10.3390/ijms23137003)
Supplement: Supplementary file 1 [file ijms-23-07003-s001.zip › ijms-1779308-supplementary.pdf]

Supplementary Materials

The 2-hydroxy-3-(4-aryl-1-piperazinyl)propyl Phthalimide Derivatives as Prodrugs—Spectroscopic and Theoretical Binding Studies with Plasma Proteins

Aleksandra Marciniak [1,*], Aleksandra Kotynia [1], Dominika Szkatuła [2], Edward Krzyżak [1]

[1] Department of Inorganic Chemistry, Wroclaw Medical University, ul. Borowska 211a, 50-556 Wrocław, Poland; aleksandra.kotynia@umw.edu.pl (A.K.); edward.krzyzak@umw.edu.pl (E.K.)
[2] Department of Medicinal Chemistry, Wroclaw Medical University, ul. Borowska 211, 50-556 Wroclaw, Poland; dominika.szkatula@umw.edu.pl
* Correspondence: aleksandra.marciniak@umw.edu.pl; Tel.: +48-71-784-03-35

Supplementary materials

List of Supplementary Files:

1. Supplementary Figures S1-S2

2. Supplementary Tables S1-S5

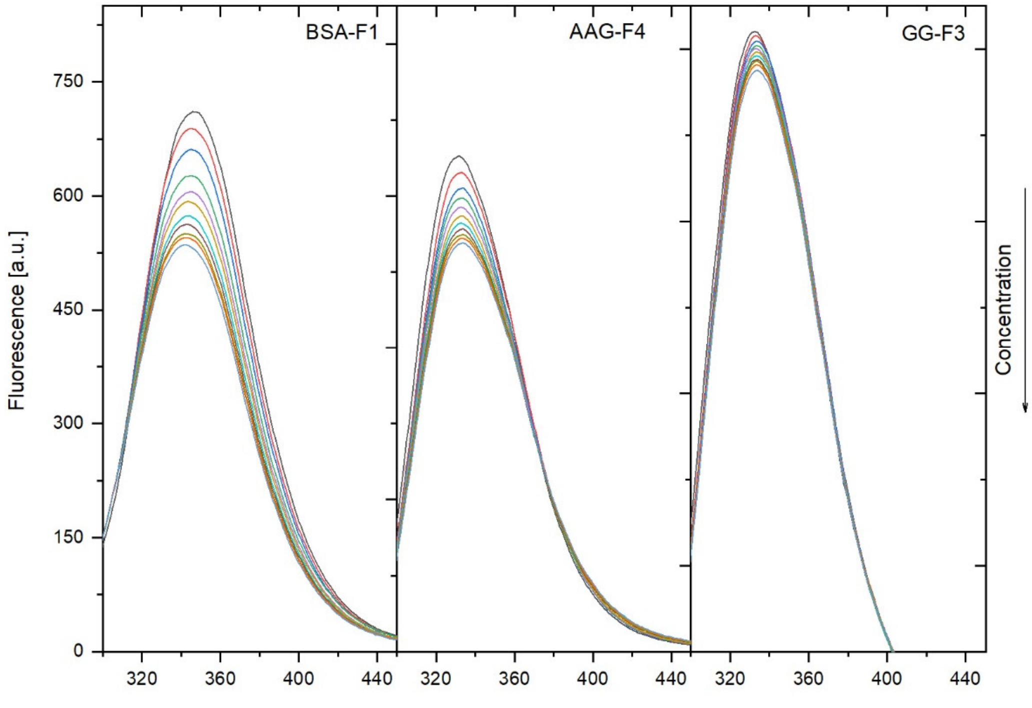

Figure S1. Fluorescence spectra of BSA-F1, AAG-F4, and GG-F3 systems in the presence of different concentration of compounds F1, F4, and F3.

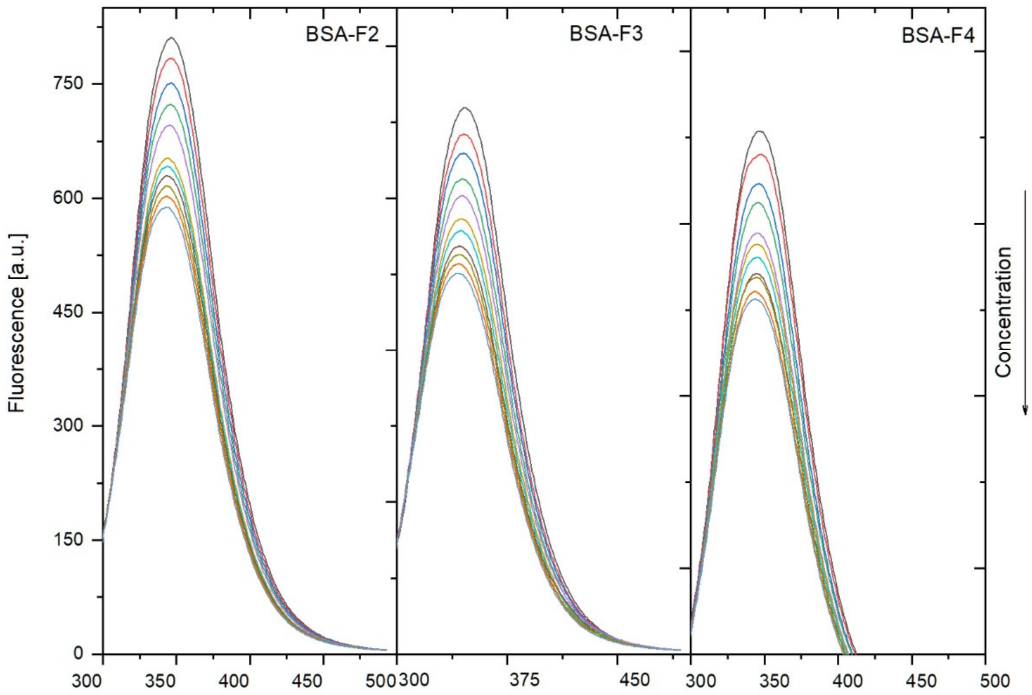

Figure S2. Fluorescence spectra of BSA-F2, BSA-F2, and BSA-F3 systems in the presence of different concentration of compounds F2-F4.

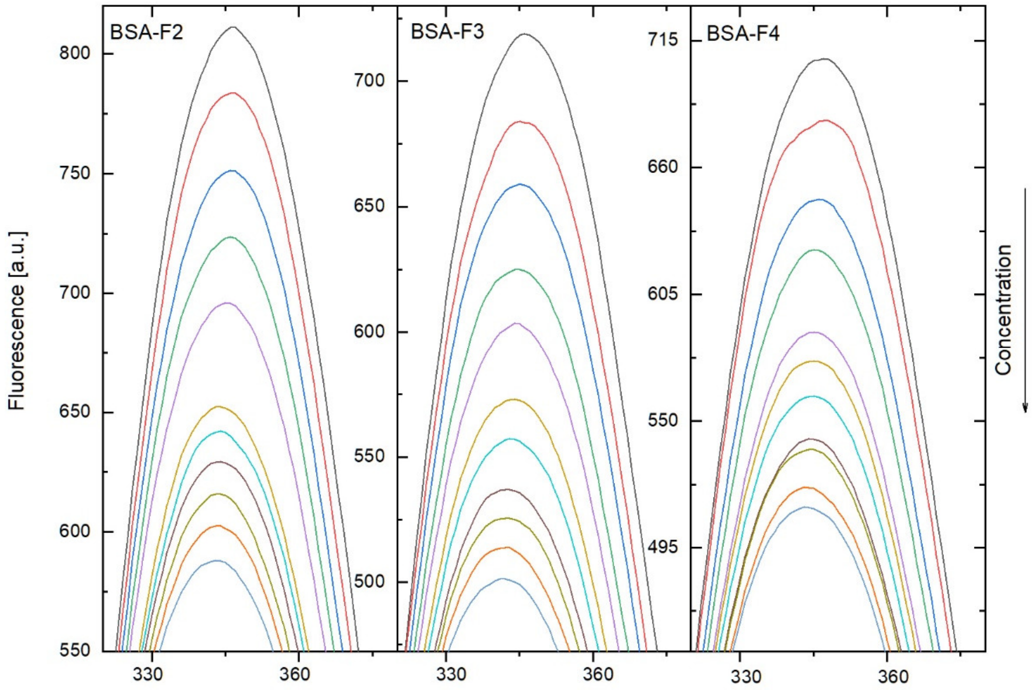

Figure S3. Fluorescence spectra (zoom on maximum) of BSA-F2, BSA-F2, and BSA-F3 systems in the presence of different concentration of compounds F2-F4.

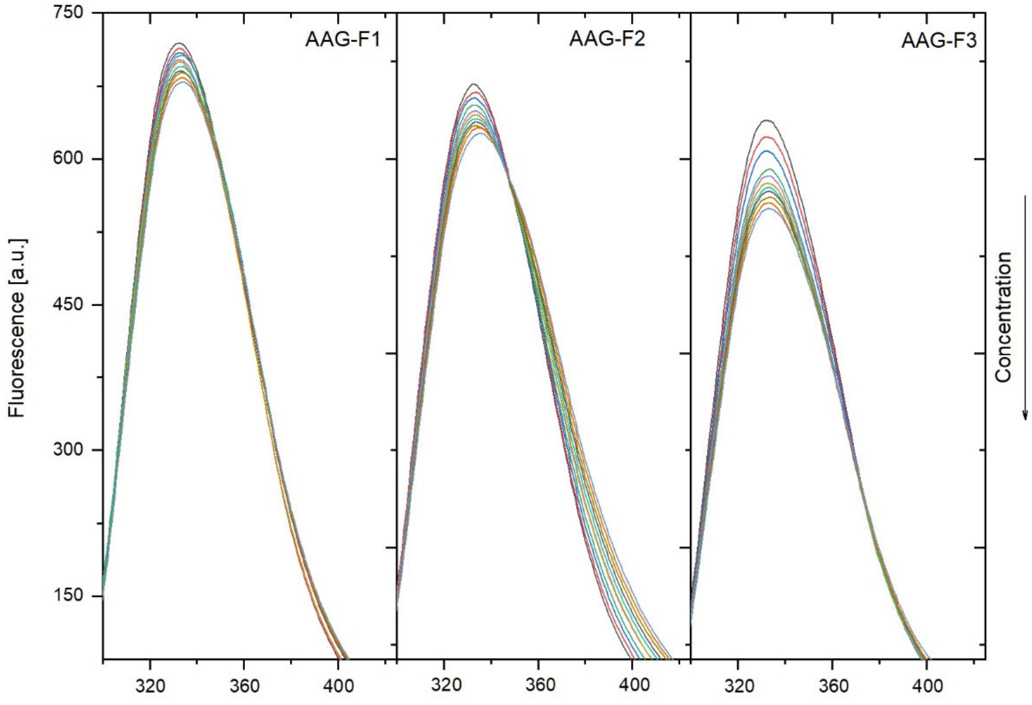

Figure S4. Fluorescence spectra of AAG-F1, AAG-F2, and AAG-F3 systems in the presence of different concentration of compounds F1-F3.

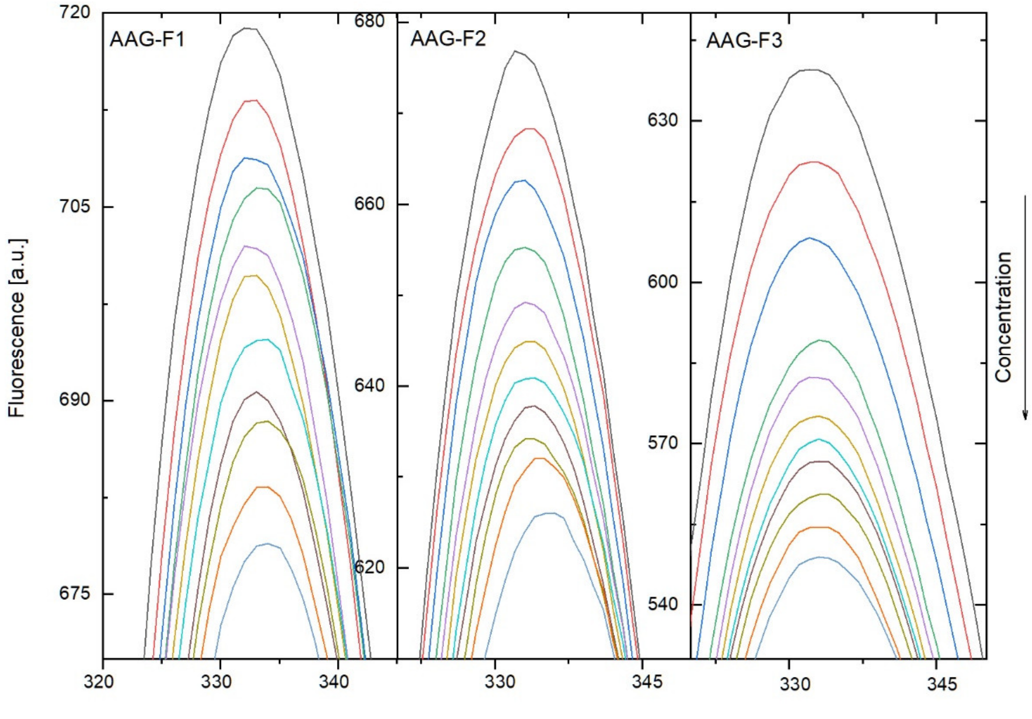

Figure S5. Fluorescence spectra (zoom on maximum)of AAG-F1, AAG-F2, and AAG-F3 systems in the presence of different concentration of compounds F1-F3.

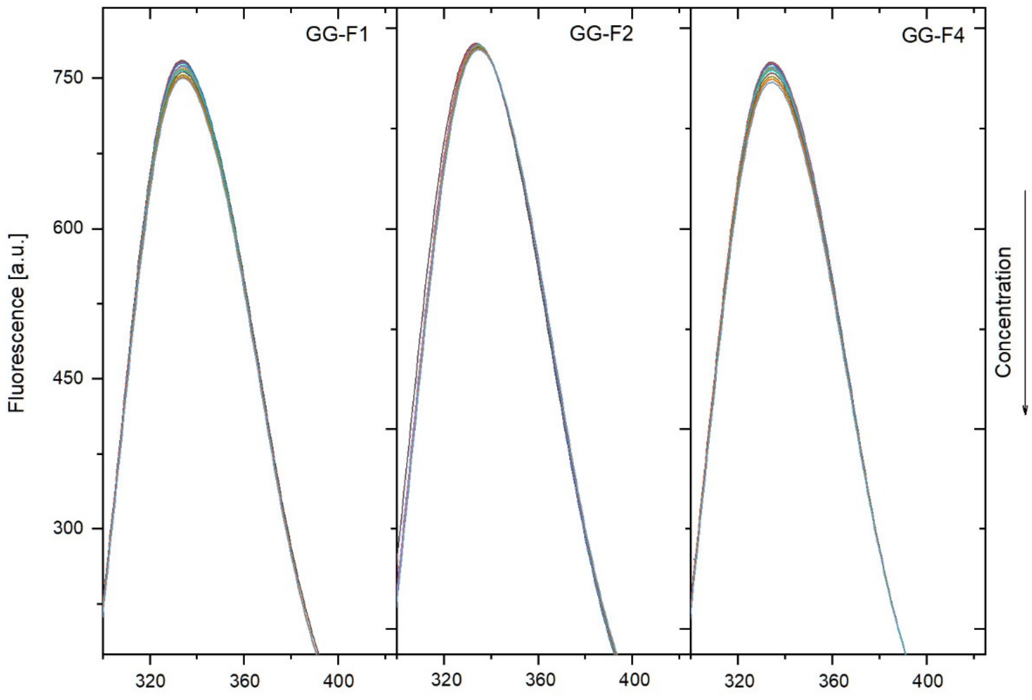

Figure S6. Fluorescence spectra of GG-F1, GG-F2, and GG-F4 systems in the presence of different concentration of compounds F1, F2, F4.

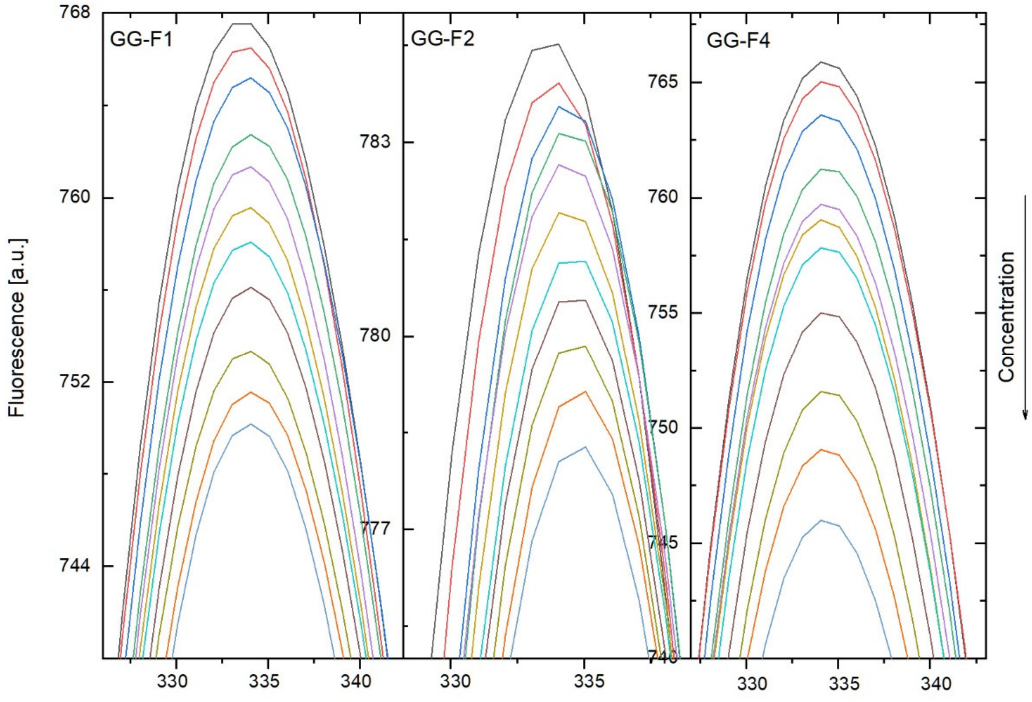

Figure S7. Fluorescence spectra(zoom on maximum) of GG-F1, GG-F2, and GG-F4 systems in the presence of different concentration of compounds F1, F2, F4.

Table S1. The percentage of content of secondary structure elements in BSA in the absence and presence of compounds F1, F2, F3, and F4.

BSA : analyzed compound molar ratio	% α-helix	% β-sheet	% β-turn	% other
**Compound F1**				
1:0	58.6%	6.1%	10.0%	25.3%
1:0.5	58.0%	6.8%	10.0%	25.2%
1:1	57.7%	6.9%	10.0%	25.3%
1:2	57.6%	7.4%	10.0%	25.0%
1:5	57.2%	7.6%	10.0%	25.2%
1:10	56.7%	8.0%	10.1%	25.3%
**Compound F2**				
1:0	60.4%	5.2%	9.9%	24.5%
1:0.5	59.8%	5.4%	10.0%	24.9%
1:1	59.3%	5.7%	10.0%	25.0%
1:2	59.0%	5.9%	10.0%	25.0%
1:5	58.5%	6.2%	10.1%	25.2%
1:10	57.9%	7.0%	10.0%	25.1%
**Compound F3**				
1:0	60.5%	5.2%	9.9%	24.4%
1:0.5	59.8%	5.9%	9.9%	24.4%
1:1	59.3%	6.4%	9.9%	24.4%
1:2	59.1%	6.6%	9.9%	24.4%
1:5	58.6%	6.9%	9.9%	24.6%
1:10	57.9%	7.1%	10.0%	25.0%
**Compound F4**				
1:0	60.1%	5.5%	9.9%	24.5%
1:0.5	59.5%	6.5%	9.8%	24.2%
1:1	58.9%	6.9%	9.9%	24.3%
1:2	58.5%	7.2%	9.9%	24.4%
1:5	58.2%	7.6%	9.9%	24.3%
1:10	57.7%	8.0%	9.9%	24.4%

Table S2. The percentage of content of secondary structure elements in AAG in the absence and presence of compounds F1, F2, F3, and F4.

AAG : analyzed compound molar ratio	% α-helix	% β-sheet	% β-turn	% other
**Compound F1**				
1:0	35.1%	29.2%	9.5%	26.3%
1:0.5	34.6%	29.3%	9.5%	26.6%
1:1	34.3%	29.2%	9.5%	27.0%
1:2	34.1%	29.5%	9.5%	26.9%
1:5	33.8%	29.4%	9.6%	27.2%
1:10	33.4%	29.7%	9.6%	27.3%
**Compound F2**				
1:0	33.7%	29.6%	9.6%	27.1%
1:0.5	33.3%	29.8%	9.6%	27.3%
1:1	33.2%	29.8%	9.6%	27.5%
1:2	33.1%	30.1%	9.6%	27.2%
1:5	32.9%	30.3%	9.6%	27.2%
1:10	32.4%	30.4%	9.7%	27.6%
**Compound F3**				
1:0	33.7%	29.8%	9.5%	27.0%
1:0.5	33.1%	29.8%	9.6%	27.5%
1:1	33.3%	29.9%	9.6%	27.2%
1:2	33.1%	29.6%	9.7%	27.7%
1:5	32.6%	30.3%	9.7%	27.5%
1:10	33.7%	29.8%	9.5%	27.0%
**Compound F4**				
1:0	33.5%	29.5%	9.6%	27.5%
1:0.5	33.3%	29.5%	9.6%	27.5%
1:1	33.0%	29.5%	9.6%	27.8%
1:2	32.9%	30.1%	9.6%	27.4%
1:5	32.5%	29.9%	9.7%	27.9%
1:10	31.8%	30.5%	9.7%	28.0%

Table S3. The percentage of content of secondary structure elements in GG in the absence and presence of compounds F1, F2, F3, and F4.

GG: analyzed compound molar ratio	% α-helix	% β-sheet	% β-turn	% other
**Compound F1**				
1:0	10.0%	37.8%	13.3%	38.8%
1:0.5	9.9%	38.0%	13.3%	38.8%
1:1	9.8%	37.8%	13.3%	39.1%
1:2	9.9%	38.0%	13.3%	38.8%
1:5	9.7%	38.2%	13.3%	38.8%
1:10	9.4%	38.8%	13.2%	38.5%
**Compound F2**				
1:0	9.7%	38.3%	13.3%	38.7%
1:0.5	9.4%	38.0%	13.3%	39.2%
1:1	9.5%	38.3%	13.3%	38.9%
1:2	9.4%	38.1%	13.3%	39.1%
1:5	9.4%	38.1%	13.3%	39.1%
1:10	9.2%	39.3%	13.2%	38.2%
**Compound F3**				
1:0	9.6%	37.9%	13.3%	39.1%
1:0.5	9.6%	37.9%	13.3%	39.1%
1:1	9.5%	38.3%	13.3%	38.9%
1:2	9.4%	37.9%	13.3%	39.3%
1:5	9.5%	38.6%	13.2%	38.6%
1:10	9.2%	38.3%	13.3%	39.2%
**Compound F4**				
1:0	9.7%	38.1%	13.3%	39.0%
1:0.5	9.5%	38.1%	13.3%	39.0%
1:1	9.5%	37.9%	13.3%	39.2%
1:2	9.6%	38.0%	13.3%	39.1%
1:5	9.5%	38.4%	13.3%	38.8%
1:10	9.4%	39.0%	13.2%	38.3%

Table S4. The percentage of the secondary structure of blood serum proteins BSA, AAG, GG, and complexes with compound F1-F4 at pH=7.5 with equimolar ratio, calculated from deconvolution Amide I band obtain by ATR-IR spectra.

compound	% α-helix	% β-sheet	% β-turn	% β-anti	% random
			**BSA**		
free protein	57.92%	12.67%	9.27%	9.01%	11.13%
F1	48.1%	16.71%	9.41%	9.46%	16.32%
F2	45.72%	17.18%	9.28%	11.53%	16.29%
F3	49.52%	14.51%	9.25%	10.47%	16.25%
F4	47.29%	15.82%	9.06%	11.78%	16.05%
			**AAG**		
free protein	34.75%	26.24%	8.60%	3.33%	27.08%
F1	28.67%	31.71%	10.45%	4.34%	24.83%
F2	28.26%	28.55%	10.66%	6.82%	25.71%
F3	32.11%	28.51%	7.68%	5.55%	26.51%
F4	28.73%	31.12%	8.13%	6.28%	25.74%
			**GG**		
free protein	11.56%	22.45%	20.84%	5.70%	39.45%
F1	11.39%	27.21%	19.79%	2.56%	39.06%
F2	10.61%	21.78%	20.47%	2.89%	38.26%
F3	10.85%	23.99%	24.33%	2.70%	38.10%
F4	10.93%	26.29%	21.81%	3.26%	37.71%
